# Acute respiratory infection related to air pollution in Bamenda, North West Region of Cameroon

**DOI:** 10.11604/pamj.2019.32.99.15228

**Published:** 2019-03-04

**Authors:** Marius Nsoh, Bassong Olga Yvonne Mankollo, Mbondji Ebongue, Kengne Nde Cyprien, Julienne Louise Ngo Likeng, Sheikh Mohammed Shariful Islam, Andrew Collier, Joyce Mahlako Tsoka-Gwegweni, Samuel Nambile Cumber

**Affiliations:** 1 School of Health Sciences, Department of Public Health, Catholic University of Central Africa, Yaounde, Cameroon; 2 School of Health Systems and Public Health, University of Pretoria, Pretoria, South Africa; 3 Bordeaux School of Public Health and Epidemiology University of Bordeaux, Bordeaux, France; 4 Cardiovascular Division, the George Institute for Global Health, University of Sydney, Sydney, Australia; 5 University Hospital Ayr, Ayr, United Kingdom; 6 School of Nursing & Public Health, College of Health Sciences, University of KwaZulu-Natal, Durban, South Africa; 7 Faculty of Health Sciences, University of the Free State, Bloemfontein, South Africa; 8 Section for Epidemiology and Social Medicine, Department of Public Health, Institute of Medicine (EPSO), The Sahlgrenska Academy at University of Gothenburg, Gothenburg, Sweden

**Keywords:** Ambient pollution, indoor pollution, risk factors, morbidity, particulate matter, acute respiratory infection

## Abstract

**Introduction:**

Air pollution is a global health problem. It's responsible for over 4 million deaths each year and constitutes a risk factor for acute respiratory infections (ARI). The aims of this study was to assess knowledge about air pollution, and to determine environmental risk factors associated with ARIs occurence in the city of Bamenda, Cameroon.

**Methods:**

We conducted a cross sectional study and performed a rectrospective analysis of ARI consultation within the period March 2016 to July 2016 in the Bamenda Health District. We interviewd 201 patients and recorded 1849 cases from hospital registers of patients diagnosed ARI from January 2013 to April 2016. Epi-info 7.2 was used for data entry and analysis. Logistic regression analysis was conducted to determine the importance of the different environmental risk factors.

**Results:**

Over 70% of the participants used at least a form of solid fuel for cooking. The Odds of developing an ARI was 3.62 greater among those exposed to indoor cooking compared to the unexposed (OR 3.62, CI 1.45-4.90). Participants exposed to open fire burning were 1.91 times more like to develop ARI compared to unexposed (OR: 1.91, CI 1.03-3.55: p : 0.03). Particulate Matter (PM 2.5) levels was 13.2 times higher than the World Health Organization (WHO) recommended levels. Dry and dusty weathers increased the risk of ARIs (OR 3.24; CI 1.47-7.13). The prevalence of ARIs in the Bamenda Health District was 6% of all consultations.

**Conclusion:**

Using solid fuels in poorly ventilated homes increase the total air particle suspension indoor. Inhalling this poor air irritates the repiratory tract, eyes while longterm exposure increases the odds of cancers. Ventilating homes with indoor cooking space reduces exposure while using clean fuels like electricity reduces the odds of ARI associated with pollution.

## Introduction

**Figure 1:**
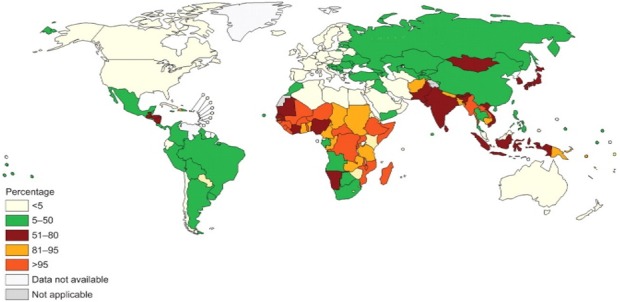
World's population using solid fuels expressed in percentage (2012)

In many developing countries, pollution constitutes a major risk factors associated with increased adverse health effects. Indoor pollution contributes significantly to increase morbidity and mortality of disease [1]. A large portion of the world's population still rely on solid fuel for cooking, including crop residues, firewood and charcoal [2]. These solid fuels undergo incomplete burning and release substantial amounts of toxic tiny respirable particles to the atmosphere that increases the odds of respiratory system. PM is associated with a higher incidence of upper airway symptoms, such as rhinorrhea, nasal obstruction, cough, laryngospasm, and vocal fold dysfunction, and lower airway symptoms, such as cough, dyspnea, and wheezing [3] (Figure 1).

Other major sources of mobile or static particles are emitted from, forest fires, agricultural burning, home waste and plastic burning, motor vehicles exhaust fumes, smoke from power plants, industries, and antmosperic stability and wind that can influence transport and deposition of particles [4,5]. In Cameroon, approximately 7,000 deaths are associated with household air pollution annually with an average PM2.5 particles level of 65 μg/m^3^, which is 6.5 times higher than the WHO recommended safe level [6]. The odds of pollution is mostly borne by women and children who spend generally the highest time at home and in kitches compared to men. Environmental pollution is a major risk factor for acute respiratory infections (ARI) in children and a major cause of childhood mortality. However, data on environmental risk factors for ARI in many developing countries is limited [7]. The aim of this study was to determine the environmental risk factors and seasonal trends associated with ARI outcomes in Bamenda.

## Methods

We conducted a cross sectional study and performed retrospective analysis of ARI consultation within the period March 2016 to July 2016 in the Bamenda Health District. The district was stratified into two based on target populations of the entire zone. The Azire, and Nkwen Urban Integrated Health Center lead health facilities of health ares with a cumulated population of about 100,000 inhabitants were the areas of study. Data concerning age, gender, area of residence, diagnosis, and outcome were collected from hospital registers. We conceived and administed a pretested structured questionnaire to patients diagnosed from an ARI to evaluate their knowledge on pollution and odds to health. ARI was defined by the presence of fever, cough, catarrh and other presenting symptoms confirmed by a clinician. We later followed the participants to their homes to assess their household standard. Data on the size of the house, number of rooms, kitchen location and total number of inhabitants in each house were collected. An epidemiologic disease profile for ARIs was established based on the hospital registers records. Data on age, diagnosis, symptoms presented, months, year of exposure were collected. The data was entered and analysed using Epi Info version 7.2. Bivariate and Multvariate analysis performed for Logistic regression to assess association between ARI, indoor cook, season, fuel type open fire burn and second hand smoke. Data of PM2.5 was extracted from the WHO database [6].

**Ethics**: To respect the medical ethical considerations, we ensure confidentiality, justice and respect of all individuals during this research after explaining to participants the objectives of the study and the implications. A written consent was signed to before participants stated the study. This study was approved by Institutional Review Board of the School of Health Sciences, Catholic University of Central Africa No 2016/0341/CEIRSH/ESS/MSP. An authorization to conduct this research was granted by the Regional Delegate for Public Health North West Region NO59/RN/NW/RDPH. Authorization was also equally granted by the district chief of service for public health for Bamenda health district NO 18/NWR/RDPH/DHS BDA. Final approval also given by facility heads.

## Results

**Figure 2:**
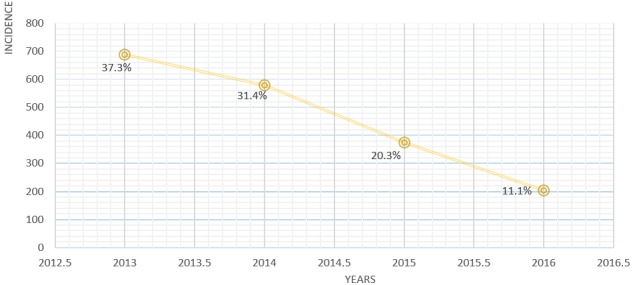
Trends of acute respiratory infections occurrence and evolution over time

**Figure 3:**
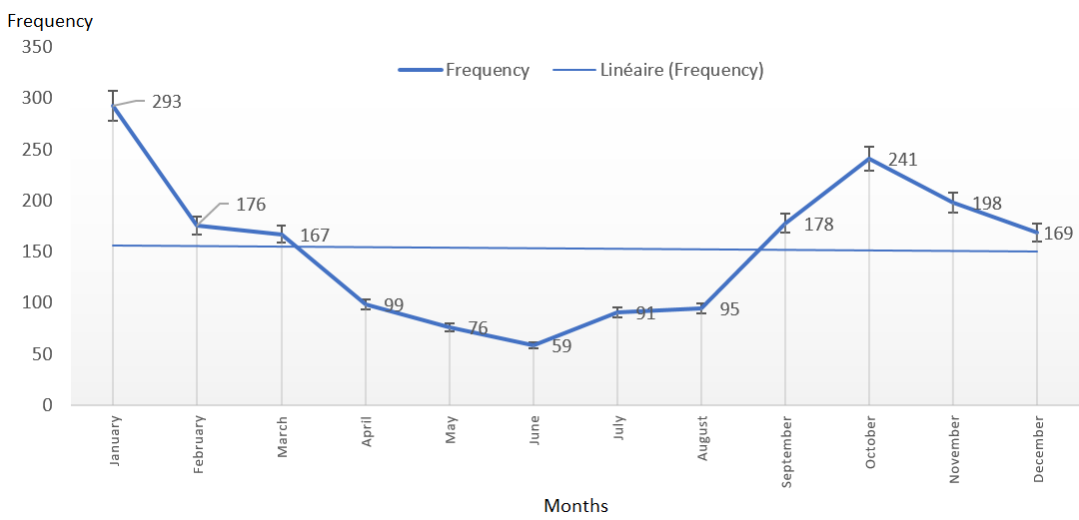
Patterns of acute respiratory infections and evolution per months of the years

There were 1849 records of ARI from hospital registers (January 2013 to December 2015). The ARIs were divided into Acute Upper Respiratory Infections (AURI) and Acute Lower Respiratory Infections (ALRI). The min age was 2 weeks and max age was 85 years, the standard deviation 13 years and mean age was 9 years meaning children are the most affected (M 51.8 v F 48.2%) 83.4% of patients consulted lived in or around the Azire health area while 16.6% consulted lived in or around the PMI Nkwen Heath area. From N 1849: 2% were ALRI while 98% were AURIs. Of the patients involved, 1054 (57%) were infants, 77 (4%) were businessmen, 567 (31%) were students, 52 (2.82%) were teachers and 95 (5.00 %) were of other professions. 688 (37.3%) cases were recorded during the year 2013, 579 (31.4%) cases in 2014, 374 (20.3%) cases in 2015 and 204 (11.1%) cases during the first quarter of 2016. We noticed a reduction of about 5.6% from 2013 to 2014 and another reduction of 11.1% from 2014 to 2015 (Figure 2, Figure 3).

October to January registered the highest incidence associated with increased temperatures of the dry season, while March to August registered a reduction in incidence with rain fall that reduced dust and bush burning. This months were characterized by extreme dryness, dusty weather with very cold mornings and very hot days. For the prospective study, 201 patients were enrolled after diagnosed to suffer from ARI by a medical doctor or senior nurse. The standard deviation was 8 years mean age two months, max age 87. The participants were enrolled after being diagnosed ARI. More than one-third of the participants (37.4%) lived in houses with five or more inhabitants. The majority of the respondents (93%) lived in houses with cemented walls and 94% lived in houses with cemented floor. About 71% households used wood as primary fuel type, 3% relied on crop residues, 59% households used liquefied petroleum gas, and 12% used kerosene as the primary fuel type. None of the households used electricity or solar energy for cooking. Most of the respondents (66 %) used indoor kitchens for cooking, 42.9% of the respondents used at least one form of chemical insect repellants indoors. More than half of the participants (61%) reported nose and/or eye irritations while cooking, 25% used toxic insect repellants and 48% houses had at least one pet animal. 36% of the respondents mentioned bush/farm burning as a major potential air pollutant. 79% considered exposure to second-hand tobacco smoke and 78% reported community plastic burning as major air pollutants. 75% reported dusty air as environmental component potentially responsible for ARIs, 69% blamed cold air, 45% cited dry air, but only 6% of the participants considered warm air as a contributor to their diseases outcome (Table 1).

**Table 1:** Unconditional logistic regressions; outdoor characteristics

Variable	Category	Yes	No	OR	P. Value
**People burn bushes around**	Yes	71.62	28.38	1.91 (1.03-3.55)	0.03*
	No	56.80	43.20		
**People burn plastics around**	Yes	65.16	51.11	1.78 (0.91-3.50)	0.08
	No	34.84	48.89		
**Second hand smoking**	Yes	65.41	34.59	1.98 (0.99-3.97)	0.05*
	No	48.78	51.22		
**Does dry air Constitute a risk?**	Yes	60.22	39.78	0.86 (0.48-1.53)	0.62
	No	63.55	36.45		
**Does cold air Constitute a risk?**	Yes	64.29	35.71		
	No	56.67	43.33		
**Does warm air Constitute a risk?**	Yes	72.72	27.27	1.67 (0.43-6.53)	0.45
	No	61.38	38.62		
**Does dusty air Constitute a risk?**	Yes	65.79	34.21	1.92 (0.99-3.77)	0.04*
	No	50.00	50.00		

*Significant variables: The particulate matter 2.5 (PM 2.5) levels were 13.2 times higher compared to the WHO recommended levels (about 10 micrograms per cubic meter) in Bamenda (132 μg/m^3^) [6] . Logistic regression analysis showed that use of one or more solid fuel types was significantly associated with ARI (OR 1.44, CI 1.21-1.92). Participants exposed to indoor cooking presented 3.6 times the odds of suffering from ARIs (OR 3.62, CI 1.45-4.90) and those exposed to environmental burning were 1.91 more likely to develop ARI (OR: 1.91, CI 1.03-3.55) compared to the non-exposed (Table 2).

**Table 2:** exposure sources and the risk of acute respiratory infection occurrence

Variable	Crude results			Adjusted results		
	OR	95% CI	P value	OR	95% CI	P value
**Sold fuel use**	1.66	(0.32-8.45)	0.53	1.44	(1.21-1.92)	0.03*
**Indoor Cooking**	2.67	(1.45-4.90)	0.00*	3.62	(1.68-7.76)	0.00*
**Environmental Plastic burn**	01.78	(0.91-3.50)	0.08	1.25	(0.44-3.55)	0.67
**Dusty air**	1.92	(0.99-3.77)	0.04	3.24	(1.47-7.13)	0.00*

* Significant values

## Discussion

The dry season constitutes a relatively hot and dry weather that favors dust particles suspended on the atmosphere OR 3.24 on exposure of dust particles during the dry season compared to rainy season hence, dust exposure raised odds of ARI compared to unexposure [8]. Symptoms such as cough, catarrh and fever were the most common symptoms presented during consultations, whereas fatigue, nasal congestion, sneezing and wheezing presented statistically significant values. People who suffered from fatigue had 5.06 more chances to develop from AURI compare to ALRIs with (95 % CI: 2.31-15.89, p-value 0.00). Solid fuel use constitutes a major risk factor for ARI and 1.5 times the odds to develop ARIs compared to those who used cleaner fuel types. Solid fuel use is associated with higher concentrations of potentially inhaled particulate matter, as compared to cleaner fuel [9] and might be responsible for the higher prevalence of ARI. These unclean fuel types increase the concentration of household air pollution. Our results are in line with previous findings from [10] that showed, solid fuel users had 1.9 times higher risk of ARI occurence compared to those using low polluting fuel types for cooking (95% CI: 1.40-2.71), and almost four times the odds of suffering from ARI compared to households using gas (95% CI: 1.54, 28.25). Another study in the western region of Cameroon revealed that women exposed to wood smoke had a significantly higher prevalence of chronic bronchitis (7.6%) compared to the alternative fuel user group (0.6%) [11]. A Nepalese study revealed that children living in households using solid fuel had 1.79 times higher odds of suffering from ARI compared to children from households using cleaner fuel like liquefied petroleum (95% CI 1.02-3.14) [3]. The high use of solid fuels might explain the high prevalence of ARIs among children in the city of Bamenda.

In this study, participants who practiced indoor cooking had 3.6 times higher odds to develop ARIs than those who practiced outdoor cooking. A previous study revealed that participants using indoor kitchens and one of the polluting solid fuel types had 3.6 more chances to develop ARIs (95 % CI 1.45-4.90:). Participants using indoor cooking with separate kitchen were at lower risk to suffer from ARI compared to those using indoor cooking with no separate kitchen (OR:1.51; 95 % CI: 1.09-2.10) [12]. In poorly ventilated houses, indoor smoke can be 100 times higher than the acceptable recommendation by the WHO [13]. Exposure is particularly high among females, children, elderly and those with ill health spending most of their time at homes. In most low-income countries, cooking and food preparation is mostly performed by females with children close by them during cooking, which can increase their exposure and risks of acute and long-term ill health linked to inhaled emissions of particles released by incomplete combustion of solid fuels [14]. Bush burning was a common practice in the study area and was associated with ARIs. Participants exposed to bush burning had almost twice the chances of suffering from an ARI symptom, which is similar to a study in Grenada. This study showed participants engaged in bush burning had a statistically significant higher prevalence of sinusitis symptoms (OR: 2.1, 95 % CI 1.1-3.9) and cough (OR: 1.6, 95 % CI: 0.9-2.8) [15]. It is clear, that individuals and local government should take actions to reduce the risk of environmental exposures to pollutants to reduce the risk of ARIs. Long-term exposure to solid fuel smoke is clearly associated with chronic obstructive pulmonary disease (COPD), increased risk of acute respiratory infections/pneumonia, lung cancer, tuberculosis (TB) and cataracts [7]. Many respiratory diseases have been found to be associated with exposure to biomass fuels. The strength of association varies for diseases like acute respiratory infections (ALRI), chronic obstructive pulmonary disease (COPD), lung cancer, pulmonary tuberculosis (TB), and asthma [16]. Results revealed that people who presented with breathlessness had 0.94 (94%) chances to be suffering from an ALRTI (OR: 0.06, p-value: 0.00). People who presented with chest pain had 0.78 (78%) chances to suffer from ALRTI (OR 0.22: p = 0.00). As concerns people who presented with fever, they had 0.75 (75%) chances to suffer from an ALRTI (OR: 0.25 p = 0.00). Those who presented with nasal congestion had 0.96 (96%) chances to suffer from an ALRTI (OR 0.4).Those who presented with sneezing had 0.97 (97%) chances to suffer from an ALRTI with (OR 0.0) and finally people who presented with wheezing had 0.99 (99%) chances to suffer from an ALRTI (OR 0.1). Sneezing was equally presented mostly with patients suffering from ALRTI being 16.49 times more present in people with lower respiratory tract infections than for those with AURTI (OR 17.49). Wheezing was also a symptom presented mostly by those who suffered from ALRTI these occurred 43.95 times more in patients with lower respiratory tract infections compared to those with AURIs. As concern the seasonality, people suffered more of ARI during the dry season with 48 times more to have the infection compared to the rainy season. The dry season constitutes a relatively hot and dry weather condition that favors dust particles that suspend on the atmosphere. The OR showed that being exposed to dust increased the risk by 3.2 time compared to non-exposed. A similar case control study conducted in Egypt to assess the effect of exposure to flour dust on respiratory symptoms and lung function of flourmill workers revealed significant results with.

## Conclusion

Household and ambient pollution remains a significant contributor to ARI in the city of Bamenda. Using solid fuels in poorly ventilated homes increase the total air particle suspension indoor. Inhaling this poor air irritates the respiratory tract, eyes, skin, while long-term exposure increases the odds of COPD, and cancers. Ventilating homes with indoor cooking space reduce exposure while using clean fuels like electricity reduces the odds of ARI associated with pollution.

### What is known about this topic

- Bamenda is one of the most polluted cities with PM2.5 It is up to 13 times exceeding WHO recommended level;
- The Bamenda health district placed ARI amongst her major public health priorities because of its high prevalence;
- Air pollution today constitutes a global thread with deadly outcomes. It is a public health emergency responsible for million deads worldwide.

### What this study adds

- Atmospheric stability and wind contributes to raise total air suspension particles in Bamenda;
- Creates an awareness of the risk factors that increases the odds of ARI through community sensitization in Bamenda;
- Recommend to switch from highly polluting solid fuels to medium liquid fuels or electricity as cleanest fuel in this communities.
